# Direct observation of procedural skills (DOPS) assessment in diagnostic gastroscopy: nationwide evidence of validity and competency development during training

**DOI:** 10.1007/s00464-019-06737-7

**Published:** 2019-03-25

**Authors:** Keith Siau, James Crossley, Paul Dunckley, Gavin Johnson, Mark Feeney, Neil D. Hawkes, Ian L. P. Beales, The Joint Advisory Group on Gastrointestinal Endoscopy (JAG)

**Affiliations:** 1 Joint Advisory Group on Gastrointestinal Endoscopy, Royal College of Physicians, London, UK; 2 Medical and Dental Sciences, University of Birmingham, Birmingham, UK; 3 Academic Unit of Medical Education, University of Sheffield, Sheffield, UK; 4 Department of Gastroenterology, Gloucestershire Hospitals NHSFT, Gloucester, UK; 5 Department of Gastroenterology, University College London Hospitals NHSFT, London, UK; 6 Department of Gastroenterology, Torbay and South Devon NHS Foundation Trust, Torquay, UK; 7 Department of Gastroenterology, Cwm Taf University Health Board, Llantrisant, UK; 8 Department of Gastroenterology, Norfolk and Norwich University Hospital, Norwich, UK

**Keywords:** Competence, Gastroscopy, OGD, Esophagogastroduodenoscopy, DOPS, Formative assessment

## Abstract

**Background:**

Validated competency assessment tools and the data supporting milestone development during gastroscopy training are lacking. We aimed to assess the validity of the formative direct observation of procedural skills (DOPS) assessment tool in diagnostic gastroscopy and study competency development using DOPS.

**Methods:**

This was a prospective multicentre (*N* = 275) analysis of formative gastroscopy DOPS assessments. Internal structure validity was tested using exploratory factor analysis and reliability estimated using generalisability theory. Item and global DOPS scores were stratified by lifetime procedure count to define learning curves, using a threshold determined from receiver operator characteristics (ROC) analysis. Multivariable binary logistic regression analysis was performed to identify independent predictors of DOPS competence.

**Results:**

In total, 10086 DOPS were submitted for 987 trainees. Exploratory factor analysis identified three distinct item groupings, representing ‘pre-procedure’, ‘technical’, and ‘post-procedure non-technical’ skills. From generalisability analyses, sources of variance in overall DOPS scores included trainee ability (31%), assessor stringency (8%), assessor subjectivity (18%), and trainee case-to-case variation (43%). The combination of three assessments from three assessors was sufficient to achieve the reliability threshold of 0.70. On ROC analysis, a mean score of 3.9 provided optimal sensitivity and specificity for determining competency. This threshold was attained in the order of ‘pre-procedure’ (100–124 procedures), ‘technical’ (150–174 procedures), ‘post-procedure non-technical’ skills (200–224 procedures), and global competency (225–249 procedures). Higher lifetime procedure count, DOPS count, surgical trainees and assessors, higher trainee seniority, and lower case difficulty were significant multivariable predictors of DOPS competence.

**Conclusion:**

This study establishes milestones for competency acquisition during gastroscopy training and provides validity and reliability evidence to support gastroscopy DOPS as a competency assessment tool.

**Electronic supplementary material:**

The online version of this article (10.1007/s00464-019-06737-7) contains supplementary material, which is available to authorised users.

Gastroscopy is the gold standard procedure for evaluating the upper gastrointestinal tract, the outcome of which is operator-dependent [1–4]. The endpoint of gastroscopy training is to ensure that trainees have developed the technical and non-technical competencies required to reliably perform the procedure without the need of supervision and to be able to accurately interpret the findings and devise a management plan. Assessments under direct observation allow for such competencies to be objectively evaluated. Formative assessments can highlight specific procedural strengths and weaknesses, allowing performance enhancing feedback and objective setting. When used sequentially, formative assessments can be used to indicate progression, consistency of performance, and readiness for summative (high-stakes) assessment, which determines suitability for independent, unsupervised practice.

Purpose-specific assessment tools are required to objectively measure competence. The gastroscopy direct observation of procedural skills (DOPS) is a formative assessment tool administered by the Joint Advisory Group on Gastrointestinal Endoscopy (JAG) [5]. The JAG oversees the governance of all gastrointestinal endoscopy training in the United Kingdom [6]. The pathway for training and certification is the same whatever the background of the trainee (gastroenterologist, surgeon, or non-medical endoscopist [NME]) and training and formative and summative assessments are performed by all trainers of all backgrounds. These are available on the JAG Endoscopy Training System (JETS) e-portfolio, a web-based platform used by all UK endoscopy trainees to prospectively record training procedures and assessments [7], which is assessed centrally to determine whether a trainee should receive certification for independent practice. The gastroscopy DOPS was designed following multidisciplinary expert consensus. It follows the standard DOPS format of deconstructing a procedure into constituent steps (items), enveloped within broader groupings (domains). The gastroscopy DOPS was modified in July 2016 to include change to the 4-point scoring scale from a performance-based to a supervision-based (entrustment) scale [8], refinement of items and descriptors (following further working group review), and a generic domain for assessing endoscopic non-technical skills (ENTS). The updated gastroscopy DOPS contains 34 items, split into six domains, with each item accompanied by a detailed descriptor (Supplementary Fig. 1) [5]. Each DOPS contains an assessor’s global rating of the overall procedure. DOPS are electronically submitted onto the e-portfolio by the assessor (trainer). Descriptors serve to standardise DOPS scoring and trainers are taught the appropriate use of the forms on gastroscopy Training the Trainers course. Engagement in DOPS has also been embedded into the UK-wide pathway for certification in diagnostic gastroscopy [9], which is mandated in gastroenterology and upper gastrointestinal surgery specialty training programmes [10, 11]. Despite this, validity and reliability evidence for gastroscopy DOPS remain lacking.

Determining when specific endoscopic competencies are likely to be attained can be insightful to trainees, trainers, and training programmes. Only two publications have evaluated competency development during gastroscopy training. The initial study by Cass et al. [12] enrolled 12 trainees and reported a successful oesophageal intubation rate of 90% at 100 procedures. More recently, a UK JETS e-portfolio study estimated that 187–200 gastroscopy procedures were necessary to achieve ≥ 95% unassisted D2 intubation rates [4]. However, beyond these single technical endpoints, the learning curves for a variety of other technical, cognitive, and non-technical competencies, e.g. pre- and post-procedural management, have not been characterised. Competence-assessment tools such as DOPS are well placed to evaluate this when studied across a large training cohort.

In this national study involving DOPS assessments of gastroscopy trainees, we aimed to (i) assess the validity and reliability of formative DOPS, (ii) use DOPS scores to evaluate competence development during training, and (iii) identify independent predictors of competence in DOPS.

## Methods

### Study design

We performed a prospective, observational, UK-wide study of all in-training formative gastroscopy DOPS submitted onto the JETS e-portfolio between July 2016 and December 2017. Under JAG recommendations, trainees perform at least one DOPS for every 10 training procedures, with the decision for DOPS to be made prior to commencing a procedure to minimise case-selection bias. For each DOPS, individual item scores, case difficulty, and assessor’s global rating were analysed. The trainee and assessor identifier, trainee grade, and lifetime procedural count immediately preceding the DOPS assessment were systematically collected.

### Study approval

Users of the JETS e-portfolio agreed to a privacy statement declaring that trainee data may be used for audit and research purposes. Formal ethics approval was not required, as the data analysed was anonymised and contained no patient identifiable data.

### Validity framework

We presented validity using Messick’s contemporary framework which proposes five sources of validity evidence in assessment tools [13, 14]: content (relevance), response process (relationship between intended construct and thought processes of assessors), internal structure (associations between test measures, i.e. reliability), relations with other variables (discriminative ability), and consequences (value implications of interpreting test scores).

### Outcomes

The following outcomes were studied in accordance to the validity framework:

1. Internal structure of DOPS: exploratory factor analysis to determine whether the distribution of scores within DOPS could suggest the assessment of different constructs. Reliability was estimated using generalisability theory (*described below*).
2. Relationship to other variables: Individual and global DOPS scores were stratified by lifetime procedure count in order to map learning curves for individual and groups of competencies. Trainee-level predictors of DOPS competence at were also studied.
3. Consequential validity: determining optimal competency thresholds with regard to overall competence.

### Statistical analyses

#### DOPS scoring

To facilitate analysis, each item scoring outcome was converted into a numerical ordinal scale, i.e. Score 1 (requiring maximal supervision), Score 2 (requiring significant supervision), Score 3 (requiring minimal supervision), Score 4 (competent for unsupervised practice). DOPS items rated N/A were excluded from item analyses. JAG arbitrarily stipulates for DOPS to be deemed competent if 90%+ of DOPS items were rated Score 4, and up to 10% as Score 3, with no Scores of 1 or 2 s, which equates to a mean score of 3.9. Receiver operating characteristics (ROC) curve analyses were performed to identify the mean score threshold which would provide optimal sensitivity and specificity in delineating overall competence.

#### Exploratory factor analysis

Exploratory factor analysis (EFA) was performed using principle axis factoring with a threshold of Eigenvalue = 1 and Varimax rotation in order to extract positively correlated factors into main groupings [15]. Sampling adequacy was tested with the Kaiser–Meyer–Olkin measure (accepted if > 0.50) and Bartlett’s test of sphericity (sufficient if *p* < 0.05). Item factor loadings > 0.4 were considered to be of good fit [16].

#### Generalisability theory

Reliability estimates were performed using generalisability theory [17, 18], a conceptual framework which applies variance component analysis to estimate the influence of key assessment variables on overall assessor rating. In this instance, those variables are trainee ability (across all assessors and cases: V_trainee_), assessor stringency (across all trainees and cases: V_assessor_), assessor subjectivity attributable to the trainee (V_assessor*trainee_), and residual variation (V_error_), most of which will be trainee case-to-case variation. From these data, generalisability coefficients (G) can be calculated as a function of the number of cases and assessors. The generalisability coefficient is based on the same general equation as a reliability coefficient (subject variance/subject variance + error variance). Like a reliability coefficient, it has a range of values between 0 (no reliability) and 1 (total reliability). A coefficient of ≥ 0.70 is the generally accepted threshold for in-training assessments.

#### Relationship with other variables

First, mean DOPS scores were calculated at item level, domain level, and for the global DOPS scores (mean item DOPS score and overall assessor rating), and grouped by lifetime procedure count to estimate learning curves for the entire cohort. Correlation analyses were made using Spearman’s rank coefficients. Next, to account for the non-independence of procedures performed by the same trainee, a multivariable binary logistic regression analysis was performed using a generalised estimating equations (GEE) method and an autoregressive (AR1) structure to identify independent relationships with overall procedural competence (i.e. overall assessor score of 4).

Statistical analyses were performed in SPSS (v24, Arkmont, NY: IBM Corp), with *p* < 0.05 indicative of significance throughout.

## Results

### Study participants

A total of 10,086 gastroscopy DOPS were completed for 987 trainees (median DOPS per trainee: 6, IQR 2–15) by 1254 assessors (median DOPS per assessor: 4, IQR 2–9). Assessments were conducted within 275 training centres. Participant characteristics are summarised in Table 1. Median lifetime procedure counts were similar between the three major trainee specialties (gastroenterology: 129; GI surgery: 138, NME: 135; *p* = 0.071). The median number of DOPS per trainee was highest in the NME specialty (12; IQR 5–23) and lowest in GI surgery trainees (5; IQR 2–9). The overall assessor DOPS ratings comprised the following: Score 1: 2.2%, Score 2: 9.5%, Score 3: 31.2%, and Score 4: 57.1%.

**Table 1:** Trainee characteristics

	Trainees (*N* = 987)	DOPS (*N* = 10,086)
Specialty		
Gastroenterology	505 (51.2%)	5282 (52.4%)
GI Surgeon	324 (32.8%)	2207 (21.9%)
NME	119 (12.1%)	2178 (21.6%)
Radiology	4 (0.4%)	41 (0.4%)
General practitioner	4 (0.4%)	42 (0.4%)
Unknown	31 (3.1%)	319 (3.2%)
Grade (gastroenterology/surgical specialties)		
Other (*LAT*/*LAS)*	36	289
Research fellow	31	262
ST3	115	1371
ST4	140	1883
ST5	127	887
ST6	104	631
ST7/8	111	744
Consultant	40	297
Associate specialist/staff grade	124	1166

ST3 refers to the first year of specialist training

*NME* non-medical endoscopist, *ST* specialist trainee, *LAT* locum appointed for training, *LAS* locum appointed for service

### Factor structure

Exploratory factor analysis (Table 2) identified three positively correlated factors whose strongest loadings correspond with the three main assessment constructs (with corresponding DOPS domains): (1) pre-procedure, (2) technical (insertion + withdrawal and visualisation), and (3) non-technical competencies (Management of Findings, Post-Procedure, ENTS). All factor loadings exceeded 0.4.

**Table 2:** Exploratory factor analysis: rotated factor matrix revealing factor structure of DOPS across the 34 DOPS items.

Rotated factor matrix^a^			
DOPS Item	Factor		
	1	2	3
Indication		0.706	
Risk		0.739	
Confirms consent		0.816	
Preparation		0.805	
Equipment check		0.772	
Sedation		0.650	
Monitoring		0.759	
Scope handling	0.742		
Angulation/tip control	0.752		
Suction/lens cleaning	0.716		
Intubation and oesophagus	0.734		
Stomach	0.770		
Second part of duodenum	0.761		
Problem solving	0.683		
Pace and progress	0.702		
Patient comfort	0.672		
Oesophagus	0.740		
Gastro-oesophageal junction	0.750		
Fundus	0.757		
Lesser curve	0.783		
Greater curve	0.778		
Incisura	0.774		
Pylorus	0.764		
First part duodenum	0.767		
Second part duodenum	0.773		
Recognition			0.660
Management			0.729
Complications			0.705
Report writing			0.675
Management plan			0.729
Communication and teamwork			0.554
Situation awareness			0.608
Leadership			0.636
Judgement and decision making			0.693

^a^Rotation converged after seven iterations

#### Sources of variance

Variance component analysis was performed to estimate the effect of key variables on the overall DOPS assessor rating. Differences in trainee ability were responsible for 31% of the variation between DOPS assessment scores, with varying assessor stringency exerting only modest influence (8%), with assessor subjectivity (18%) and case-to-case variation accounting for the majority (43%).

#### Reliability

Combining the variance estimates, the reliability of formative DOPS was modelled based on varying combinations of trainers and observations (Table 3). Nine observations (three observations each from three different assessors) provide sufficient reliability to pass the reliability threshold of 0.70. A total of 119 trainees (12.1%) met these criteria. Trainees with ≤ 2 assessors did not reach sufficient reliability thresholds even after completing up to 20 DOPS assessments per assessor.

**Table 3:** Reliability estimates (*G*-coefficients) of formative gastroscopy DOPS based on 1–6 trainers each observing 1–20 assessments

Trainers	Observations per trainer							
	1	2	3	4	5	10	15	20
1	0.31	0.40	0.44	0.46	0.47	0.51	0.52	0.53
2	0.47	0.57	0.61	0.63	0.64	0.67	0.68	0.69
3	0.57	0.66	**0.70**	**0.72**	**0.73**	**0.75**	**0.76**	**0.77**
4	0.64	**0.72**	**0.76**	**0.77**	**0.78**	**0.80**	**0.81**	**0.82**
5	0.69	**0.77**	**0.79**	**0.81**	**0.82**	**0.84**	**0.84**	**0.85**
6	**0.73**	**0.80**	**0.82**	**0.84**	**0.84**	**0.86**	**0.87**	**0.87**

*G*-coefficients of 0.70 + based on assessor and assessment combinations (indicating sufficient reliability for in-training assessment) are shown in bold

#### Competency thresholds

**Fig. 1:**
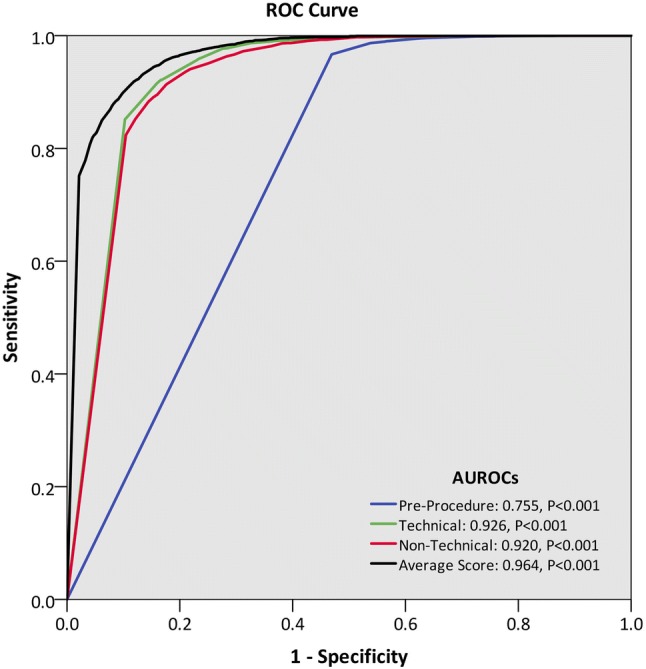
Receiver operating characteristics (ROC) curve for the ability of mean grouping scores to predict overall assessor competence. The area under the ROC (AUROC) was highest for the average (mean item) DOPS score, with a mean DOPS threshold of 3.90 providing optimal combination of sensitivity (91.9%) and specificity (88.1%)

The area under the receiver operating characteristics curve (AUROC) for mean DOPS score in predicting global competence was 0.964 (*p* < 0.001). This was higher than AUROC values for pre-procedure (0.755, *p* < 0.001), technical (0.926, *p* < 0.001), and post-procedure non-technical (0.920, *p* < 0.001) item groupings (Fig. 1). A mean DOPS cut-off score of 3.9 provided optimum sensitivity (91.9%) and specificity (88.1%) for overall competence.

#### Competency development during gastroscopy training

**Fig. 2:**
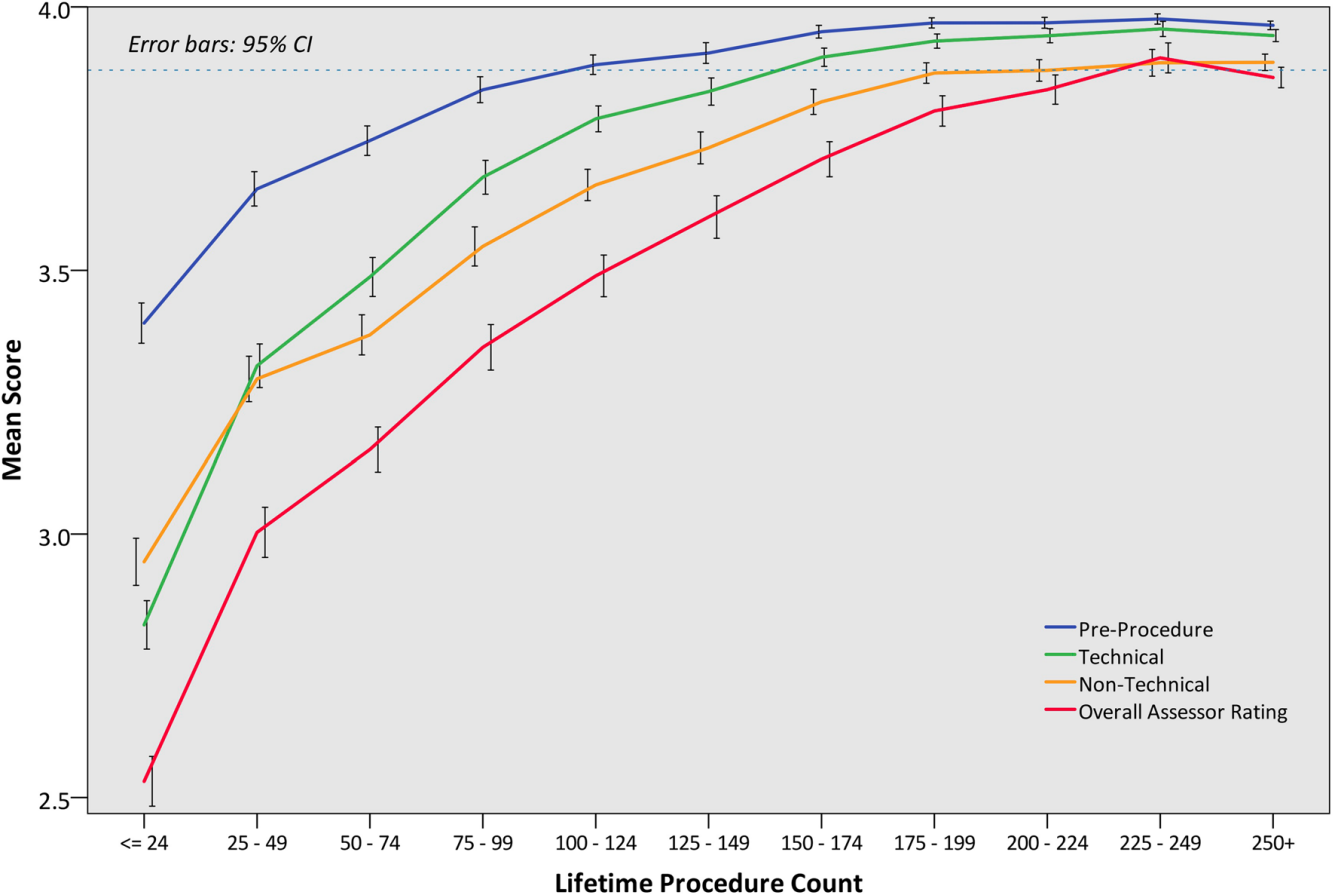
Learning curves in gastroscopy as assessed by overall DOPS scores and by the three constructs identified in factor analysis (Table 2): (a) pre-procedure, (b) technical (covering insertion and withdrawal and visualisation domains), (c) non-technical (covering management, post-procedure, and ENTS domains)

In order to illustrate learning curves across the cohort, mean DOPS scores were presented by lifetime procedure count for each item (Table 4) and domain identified from factor analysis (Fig. 2). At item level, a mean score of 3.9 was set as a competency threshold. This showed that 75–124 procedures were required to attain pre-endoscopic competencies, 150–174 procedures for technical competencies, and > 200 procedures for more advanced non-technical skills such as report writing, management plan, recognition, and management of pathology, with 225–250 procedures required to achieve global competence. Trainees acquired generic ENTS competencies in the order of “communication and teamwork” (125–149 procedures), “situation awareness”, and “leadership” (150–174 procedures), followed by “judgement and decision making” (175–199 procedures). There was positive correlation between lifetime procedural count and overall assessor rating (Spearman’s rho 0.587, *p* < 0.001).

**Table 4:** Gastroscopy DOPS performance (mean item scores) stratified by lifetime procedure count, with correlations presented as Spearman’s rho coefficients

	Lifetime Procedure Count											
	≤ 24	25–49	50–74	75–99	100–124	125–149	150–174	175–199	200–224	225–249	250 +	rho
Indication	3.4	3.6	3.7	3.8	**3.9**	**3.9**	**3.9**	**4.0**	**4.0**	**4.0**	**4.0**	0.375*
Risk	3.4	3.6	3.7	3.8	**3.9**	**3.9**	**3.9**	**4.0**	**4.0**	**4.0**	**4.0**	0.376*
Confirms consent	3.5	3.7	3.8	**3.9**	**3.9**	**3.9**	**4.0**	**4.0**	**4.0**	**4.0**	**4.0**	0.326*
Preparation	3.4	3.7	3.8	**3.9**	**3.9**	**3.9**	**4.0**	**4.0**	**4.0**	**4.0**	**4.0**	0.373*
Equipment check	3.4	3.7	3.8	**3.9**	**3.9**	**4.0**	**4.0**	**4.0**	**4.0**	**4.0**	**4.0**	0.375*
Sedation	3.3	3.5	3.6	3.8	3.8	**3.9**	**3.9**	**4.0**	**3.9**	**4.0**	**3.9**	0.388*
Monitoring	3.4	3.6	3.8	3.8	**3.9**	**3.9**	**4.0**	**4.0**	**4.0**	**4.0**	**4.0**	0.368*
Scope handling	2.8	3.3	3.5	3.7	3.8	3.8	**3.9**	**3.9**	**3.9**	**4.0**	**4.0**	0.540*
Angulation/tip control	2.7	3.2	3.4	3.6	3.8	3.8	**3.9**	**3.9**	**3.9**	**4.0**	**4.0**	0.562*
Suction/lens cleaning	2.9	3.4	3.5	3.7	3.8	**3.9**	**3.9**	**3.9**	**4.0**	**4.0**	**4.0**	0.521*
Intubation and oesophagus	2.7	3.2	3.5	3.7	3.8	3.8	**3.9**	**3.9**	**3.9**	**3.9**	**4.0**	0.533*
Stomach	2.9	3.4	3.5	3.7	3.8	**3.9**	**3.9**	**4.0**	**4.0**	**4.0**	**4.0**	0.528*
Second part of duodenum	2.7	3.2	3.4	3.6	3.8	3.8	**3.9**	**3.9**	**4.0**	**4.0**	**4.0**	0.561*
Problem solving	2.6	3.1	3.2	3.5	3.6	3.7	3.8	**3.9**	**3.9**	**3.9**	**3.9**	0.551*
Pace and progress	2.7	3.2	3.4	3.5	3.7	3.8	3.8	**3.9**	**3.9**	**3.9**	**3.9**	0.531*
Patient comfort	3.0	3.4	3.5	3.7	3.8	**3.9**	**3.9**	**3.9**	**3.9**	**3.9**	**3.9**	0.480*
Oesophagus	3.0	3.4	3.6	3.8	3.8	**3.9**	**3.9**	**4.0**	**4.0**	**4.0**	**4.0**	0.501*
Gastro-oesophageal junction	2.9	3.3	3.5	3.7	3.8	**3.9**	**3.9**	**3.9**	**4.0**	**4.0**	**3.9**	0.513*
Fundus	2.8	3.3	3.5	3.7	3.8	3.8	**3.9**	**3.9**	**4.0**	**4.0**	**4.0**	0.518*
Lesser curve	2.9	3.4	3.5	3.7	3.8	**3.9**	**3.9**	**4.0**	**4.0**	**4.0**	**4.0**	0.516*
Greater curve	2.9	3.4	3.6	3.8	3.8	**3.9**	**3.9**	**4.0**	**4.0**	**4.0**	**4.0**	0.509*
Incisura	2.9	3.3	3.5	3.7	3.8	**3.9**	**3.9**	**4.0**	**4.0**	**4.0**	**4.0**	0.513*
Pylorus	2.9	3.4	3.6	3.7	3.8	**3.9**	**3.9**	**4.0**	**4.0**	**4.0**	**4.0**	0.515*
First part duodenum	2.8	3.3	3.5	3.7	3.8	**3.9**	**3.9**	**3.9**	**3.9**	**4.0**	**4.0**	0.525*
Second part duodenum	2.7	3.3	3.5	3.7	3.8	3.8	**3.9**	**3.9**	**4.0**	**4.0**	**4.0**	0.541*
Recognition	2.7	3.1	3.2	3.5	3.6	3.7	3.7	3.8	**3.9**	**3.9**	**3.9**	0.507*
Management	2.7	3.1	3.2	3.4	3.6	3.6	3.7	3.8	3.8	**3.9**	**3.9**	0.486*
Complications	2.8	3.3	3.3	3.5	3.7	3.8	3.8	**3.9**	**3.9**	**3.9**	**3.9**	0.490*
Report writing	2.9	3.2	3.3	3.5	3.6	3.7	3.8	3.8	**3.9**	**3.9**	**3.9**	0.470*
Management plan	2.9	3.2	3.3	3.5	3.6	3.7	3.8	3.8	3.8	**3.9**	**3.9**	0.446*
Communication and teamwork	3.2	3.5	3.6	3.7	3.8	**3.9**	**3.9**	**3.9**	**3.9**	**3.9**	**3.9**	0.389*
Situation awareness	3.1	3.5	3.6	3.7	3.8	3.8	**3.9**	**3.9**	**3.9**	**3.9**	**3.9**	0.416*
Leadership	3.0	3.4	3.5	3.6	3.7	3.8	**3.9**	**3.9**	**3.9**	**3.9**	**3.9**	0.418*
Judgement and decision making	3.0	3.3	3.4	3.5	3.7	3.7	3.8	**3.9**	**3.9**	**3.9**	**3.9**	0.444*

A mean threshold of 3.9 + denotes competence

*p < 0.001

#### Predictors of DOPS competency

On multivariable analysis (Table 5), lifetime procedural count (*p* < 0.001) remained independently associated with global DOPS competence. Other trainee, trainer, and procedural factors also emerged as multivariable predictors of DOPS competence, i.e. trainee specialty (*p* = 0.028), trainee seniority (*p* = 0.011), case difficulty (*p* < 0.001), and trainer specialty (*p* = 0.002), but not the attendance of a basic skills course prior to DOPS (*p* = 0.337). Engagement in formative DOPS assessment was an independent predictor of competence (*p* < 0.001).

**Table 5:** Multivariable analysis of factors associated with competence (overall assessor score of 4) in formative gastroscopy DOPS

Factor	*N*	Multivariable Odds ratio	95% Confidence interval	*p* value
Specialty^a^				**0.028**
Gastroenterology	5269	Ref		
GI surgeon	2109	1.46	1.03–2.05	**0.031**
Non-medical endoscopist	2023	0.55	0.36–0.84	**0.006**
Radiology	41	1.24	0.06–25.1	0.888
General practitioner	42	0.31	0.08–1.14	0.077
Grade (gastro/surgical specialties)				**0.011**
Junior	4383 (46.2%)	Ref		
Senior	2794 (29.5%)	1.60	1.17–2.18	**0.003**
Non-medical endoscopist	2023 (21.3%)	NA^b^		
Other (Research Fellow)	280 (3.0%)	1.34	0.74–2.42	0.330
Lifetime procedural count				< **0.001**
<50	2210 (23.3%)	Ref		
50–99	1544 (16.3%)	1.91	1.49–2.45	< **0.001**
100–149	1486 (15.7%)	3.98	2.98–5.31	< **0.001**
150–199	1507 (15.9%)	7.32	5.23–10.2	< **0.001**
200–249	1220 (12.9%)	16.7	11.2–24.8	< **0.001**
250+	1513 (16.0%)	18.9	11.8–30.3	< **0.001**
Assessor role^a^				**0.002**
Gastroenterologist	4709 (49.7%)	Ref		
GI surgeon	1524 (16.1%)	1.77	1.30–2.43	< **0.001**
Non-medical endoscopist	3173 (33.5%)	1.40	1.08–1.80	**0.011**
General practitioner	69 (0.7%)	1.46	0.20–10.5	0.705
Case difficulty				< **0.001**
Easy	3877 (40.9%)	Ref		
Moderate	5032 (53.1%)	0.80	0.47–0.77	< **0.001**
Complicated	571 (6.0%)	0.60	0.72–0.89	< **0.001**
JAG upper gi basic skills course attendance				
No	5147 (54.3%)	Ref		
Yes	4333 (45.7%)	0.90	0.72–1.12	0.337
Lifetime DOPS count				< **0.001**
<5	2185 (23.0%)	Ref		
5–9	1656 (17.5%)	1.37	1.13–1.66	**0.001**
10–14	1530 (16.1%)	1.65	1.29–2.11	< **0.001**
15–19	1348 (14.2%)	2.29	1.72–3.04	< **0.001**
20+	2761 (29.1%)	2.99	2.11–4.24	< **0.001**

Bold *p* values are significant at *p* < 0.05

Generalised estimating equations were used to account for the non-independence of repeat DOPS by the same trainee.

^a^Excludes DOPS where data were unavailable

^b^The model assigned a coefficient of zero to prevent multicollinearity, since all NMEs were also in the NME category of the Trainee Role variable

## Discussion

In line with global reforms in medical education, training in endoscopy has largely evolved from apprenticeship models, with reliance on training time, procedural numbers, and mentor endorsement, to competency-based training, which places emphasis on the continuous assessment of competence, and to ensure milestones for competency development are met during training. In this UK-wide study involving 987 trainees from 275 UK centres, we provide validity and reliability evidence to support the use of gastroscopy DOPS as an in-training competency assessment tool. Moreover, analysis of standardised assessment data has provided insights into competency development within a national cohort of gastroscopy trainees.

In order to provide valid measurements of competence, valid and purpose-specific assessment tools are required [19]. Validity refers to how well-grounded assessments are in its purpose, whereas reliability is a component within validity which refers to the consistency of test scores awarded by an assessor. Based on Messick’s validity framework, we present internal structure evidence through generalisability theory models and factor structure analyses which compartmentalise DOPS into 3 distinct constructs, i.e. ‘pre-procedure’, ‘technical’, and ‘post-procedure non-technical’ groupings, which broadly correspond to existing domains within DOPS. Further, generalisability analyses to identify sources of variance have provided information on reliability. Relationship with other variables, i.e. discriminative validity, is shown in the correlations between different components of DOPS and lifetime procedure count, which remains significant after multivariable analyses to account for confounding factors. Consequential validity is evidenced by the use of cut-points from ROC analyses and between trainees attaining the minimum certification requirement of 200 procedures and those who have not. Content validity may be inferred from the expert multidisciplinary nature of DOPS implementation [19], whereas response process evidence may be generalised from colonoscopy DOPS [20], where trainees and trainers have previously expressed high satisfaction and confidence in standards set by DOPS.

In contrast to colonoscopy training where there is a plethora of literature on assessment tools [21, 22] and milestone acquisition [23, 24], equivalent research in gastroscopy training remains lacking. This is important as training in gastroscopy often precedes training in other endoscopic modalities. To our knowledge, only two other gastroscopy-specific assessment tools exist: the GAGES-UE [25] and ACE-EGD [26]. The GAGES-UE comprises five items: oesophageal intubation, scope navigation, ability to keep a clear endoscopic field, instrumentation, and quality of examination, but is limited by the lack of reliability data and the capacity to measure non-technical skills [25]. The ACE-EGD consists of seven items and two global (technical and cognitive) scores [26], which includes assessment of non-technical competencies, i.e. indication, pathology identification and interpretation, and an overall rating for cognitive skills, but currently lacks validity data. In comparison, the 34-item gastroscopy DOPS covers a wide breadth of technical and non-technical elements, thereby providing granularity of assessment outcomes.

Analyses of DOPS scores allows learning curves across a national cohort to be characterised for each assessed competency. Competency development in the order of pre-procedural, followed by technical and non-technical post-procedural domains, suggests that higher non-technical skills mature only upon consolidation of technical skills. This is perhaps unsurprising as attributes such as judgement and decision making, particularly in complex cases, are advanced skills which require breadth of knowledge and experience. Trainees achieved the competency threshold for D2 intubation and retroversion (visualisation of the gastric fundus) at 150–174 procedures, which is comparable to the 187 procedures required to attain 95% D2 intubation [4]. However, competencies in lesion recognition, management planning, and report writing were established only after 200 + procedures, with overall procedural competency awarded after 225–249 procedures. Our data may aid training programmes to plan training and set competency milestones, and inform on the optimal timing of training interventions, e.g. pre-clinical knowledge and simulation-based training, which has the potential to accelerate learning curves [27], and the appropriateness of minimum procedure numbers in established credentialing pathways. Indeed, the 200-procedure threshold set within the JAG certification criteria per se may not be sufficient to ensure competence in non-technical skills; this is supported by recent data which showed that UK trainees had recorded a median of 282 lifetime procedures (IQR 237–411) at the time of gastroscopy certification [28]. Increasing the minimum procedural threshold may not be the solution, as this has the potential to penalise those who acquire competency earlier [29]. The optimum minimum procedure count for competency remains open for debate, but is somewhat dependent on whether training programmes mandate the use of validated and objective assessments, e.g. DOPS, or minimal key performance indicator criteria, e.g. 95%+ D2 intubation rates, to determine and safeguard trainee competence.

Within the paradigm of competency-based education, ensuring that assessments are completed objectively and consistently is key for quality assuring training. Like all workplace based assessments, DOPS scores are influenced by case-to-case variation in trainee performance, assessor stringency (or leniency), and assessor subjectivity. The generalisability analysis shows that with appropriate sampling, good levels of reliability can be achieved. The JAG certification criteria specify a minimum of 20 DOPS during gastroscopy training, of which trainees must score competent in the last 4 of the latest DOPS as a criterion to trigger summative assessment [9]. We show that it is possible to meet reliability thresholds with 20 DOPS, provided that these have been performed by at least three different assessors. For instance, even trainees with 40 DOPS assessments from 20 observations from two different assessors would fall short of the in-training reliability threshold of 0.7. Assessor stringency and assessor variation accounted for 8% and 18% of DOPS score variance, with multivariable analysis confirming assessor specialty to be an independent predictor of DOPS competence. Compared to gastroenterologists, GI surgeon and NME assessors were more likely to award the overall competent outcome. Heterogeneity in its real-world application may be considered a limitation of the DOPS instrument, which suggests the need for further training of assessors to score performance reliably, e.g. in Train-the-Trainer courses. Moreover, only 12.1% of trainees fulfilled the reliability threshold combination of ≥ 3 DOPS each from ≥ 3 different assessors. As such, mandating at least three assessors during the latter stages of gastroscopy training would provide greater validity of formative assessment by enhancing the reliability of inferences of competency.

Other limitations should be acknowledged. First, this study was centred on DOPS which were completed during the in-training stage, rather than of summative assessment, where reliability thresholds of ≥ 0.8 are considered acceptable [14]. Outcomes in relation to key performance indicators (e.g. D2 intubation rates) and assessor feedback were not studied. Second, lifetime procedure count was based on trainee entries on the JETS e-portfolio, which is susceptible to selection bias. As assessments are usually performed by a regular trainer, there is also potential for assessor bias. Third, exploratory factor analysis may be biased owing to the pre-existing layout of domains within DOPS. Finally, our study was performed within the UK national training programme, which may challenge its generalisability. Despite this, our study includes real-world data from a large number of trainees and trainers, which provides generalisability in terms of landmarks of skills acquisition, procedural numbers, and the numbers of formative assessments and assessors required to appropriately support training, which may be of value to other countries with different training and assessment formats.

Our study provides validity evidence in support of DOPS during gastroscopy training. In addition, the observation that lifetime DOPS count was independently associated with DOPS competence suggests that proactive engagement with the formative assessment process may expedite the learning curve to competency, although this merits further study. DOPS enables trainers to identify steps which deviate from optimal practice or require improvement, and serve as a platform for high-performance feedback, which has been shown to benefit learning [27]. Furthermore, engagement with DOPS, particularly with larger numbers of assessors, confers the potential for personalised learning curves and for progression during training to be benchmarked against national data. Competency development, as measured by DOPS, can be monitored by trainees, trainers, and training programmes. The DOPS assessment appears to be both sufficiently detailed to focus on specific competency items and manageable enough to incorporate into everyday gastroscopy training. We confirmed that the acquisition of technical skills occurred much earlier in training than global competence, which requires proficiency in more complex aspects of report writing and clinical decision making, and may not be achievable by 200 lifetime procedures. Ostensibly, the JAG certification process needs to ensure an evidence-based approach to training and that the nature and timing of assessments fulfils reliability criteria. The use of evidence generated from DOPS and its potential implications on national gastroscopy training are subject to ongoing review by the JAG Quality Assurance of Training Working Group.

## Conclusion

This study establishes competencies benchmarks during gastroscopy training and provides validity and reliability evidence to support gastroscopy DOPS as an in-training competency assessment tool.

## Electronic supplementary material

Below is the link to the electronic supplementary material.

Supplementary material 1 (DOCX 31 KB)
