# Supplementary material for: Direct observation of procedural skills (DOPS) assessment in diagnostic gastroscopy: nationwide evidence of validity and competency development during training
Source: Surg Endosc. 2019 Mar 25;34(1):105–14. doi: 10.1007/s00464-019-06737-7 (PMC6946748; doi:10.1007/s00464-019-06737-7)
Supplement: Supplementary file 1 — Supplementary material 1 (DOCX 31 KB) [file 464_2019_6737_MOESM1_ESM.docx]

| **Date of procedure** |  | |  | |  |
| --- | --- | --- | --- | --- | --- |
| **Trainee name** |  | | **Membership no. (eg.**  **GMC/NMC)** | |  |
| **Trainer name** |  | | **Membership no. (eg.**  **GMC/NMC)** | |  |
| **Outline of case** |  | |  | |  |
| **Difficulty of case** | **Easy** |  | **Moderate** |  | **Complicated** |
| **Please tick appropriate box** |  |  |  |  |  |

| **Level of supervision**  Complete DOPS form by ticking box to indicate the appropriate level of supervision required for each item below. Constructive feedback is key to this tool  assisting in skill development. | **Maximal supervision**  Supervisor undertakes the majority of the tasks/decisions & delivers constant verbal prompts | **Significant supervision**  Trainee undertakes tasks requiring frequent supervisor input and verbal  prompts | **Minimal supervision**  Trainee undertakes tasks requiring occasional supervisor input and verbal  prompts | **Competent for independent practice**  no supervision required | **Not applicable** |
| --- | --- | --- | --- | --- | --- |
|  | **Pre-procedure** | | | | |
| **Indication** |  |  |  |  |  |
| **Risk** |  |  |  |  |  |
| **Confirms consent** |  |  |  |  |  |
| **Preparation** |  |  |  |  |  |
| **Equipment check** |  |  |  |  |  |
| **Sedation** |  |  |  |  |  |
| **Monitoring** |  |  |  |  |  |
| **Comments** | | | | | |
|  | **Insertion and withdrawal** | | | | |
| **Scope handling** |  |  |  |  |  |
| **Angulation / tip control** |  |  |  |  |  |
| **Suction/air/lens**  **cleaning** |  |  |  |  |  |
| **Intubation and**  **oesophagus** |  |  |  |  |  |
| **Stomach** |  |  |  |  |  |
| **2^nd^ part of duodenum** |  |  |  |  |  |
| **Problem solving** |  |  |  |  |  |
| **Pace and Progress** |  |  |  |  |  |
| **Patient Comfort** |  |  |  |  |  |
| **Comments** | | | | | |
|  | **Visualisation** | | | | |
| **Oesophagus** |  |  |  |  |  |
| **Gastro-oesophageal**  **junction** |  |  |  |  |  |
| **Fundus** |  |  |  |  |  |

Formative DOPS_Upper GI (OGD).docx Date Last updated - 01 August 2016

© Royal College of Physicians, London 2016. All rights reserved Page 1 of 5

| **Level of supervision** | | **Maximal supervision** | | **Significant supervision** | **Minimal supervision** | | **Competent for independent**  **practice** | | **Not applicable** |
| --- | --- | --- | --- | --- | --- | --- | --- | --- | --- |
| **Lesser curve** | |  | |  |  | |  | |  |
| **Greater curve** | |  | |  |  | |  | |  |
| **Incisura** | |  | |  |  | |  | |  |
| **Pylorus** | |  | |  |  | |  | |  |
| **1^st^ part duodenum** | |  | |  |  | |  | |  |
| **2^nd^ part duodenum** | |  | |  |  | |  | |  |
| **Comments** | | | | | | | | | |
| **Management of Findings** | | | | | | | | | |
| **Recognition** | |  | |  |  | |  | |  |
| **Management** | |  | |  |  | |  | |  |
| **Complications** | |  | |  |  | |  | |  |
| **Comments** | | | | | | | | | |
| **Post-procedure** | | | | | | | | | |
| **Report writing** | |  | |  |  | |  | |  |
| **Management plan** | |  | |  |  | |  | |  |
| **Comments** | | | | | | | | | |
|  | | **ENTS (endoscopic non-technical skills)** | | | | | | | |
| **Communication and**  **teamwork** | |  | |  |  | |  | |  |
| **Situation awareness** | |  | |  |  | |  | |  |
| **Leadership** | |  | |  |  | |  | |  |
| **Judgement and decision**  **making** | |  | |  |  | |  | |  |
| **Comments** | | | | | | | | | |
| The o | **Learning Objectives for the next case**  jectives should be added to the trainee’s personal development plan (PDP) once DOPS is completed | | | | | | | | |
| **1.** |  | | | | | | | | |
| **2.** |  | | | | | | | | |
| **3.** |  | | | | | | | | |
| **Overall Degree of Supervision required** | **Maximal Supervision**  Supervisor undertakes the majority of the tasks/decisions & delivers constant verbal prompts | | **Significant Supervision**  Trainee undertakes tasks requiring frequent supervisor input and verbal prompts | | | **Minimal Supervision**  Trainee undertakes tasks requiring occasional supervisor input and verbal prompts | | **Competent for independent practice**  no supervision required | |
| **Please tick**  **appropriate box** |  | |  | | |  | |  | |

b

Formative DOPS_Upper GI (OGD).docx Date Last updated - 01 August 2016

© Royal College of Physicians, London 2016. All rights reserved Page 2 of 5

# DOPS form descriptors

| **Pre Procedure** | |
| --- | --- |
| **Indication** | - Assesses the appropriateness of the procedure and considers possible alternatives |
| **Risk assessment** | - Assesses co-morbidity including drug history - Assesses any procedure related risks relevant to patient - Takes appropriate action to minimise any risks |
| **Confirms Consent** | - Early in training the consent process should be witnessed by the trainer, once competent it is acceptable for the trainee to confirm that valid consent has been gained by another trained person. - During the summative DOPS the process of obtaining consent should witnessed and assessed - Complete and full explanation of the procedure including proportionate risks and consequences without any significant omissions and individualised to the patient - Avoids the use of jargon - Does not raise any concerns unduly - Gives an opportunity for patient to ask questions by adopting appropriate verbal and non-verbal behaviours - Develops rapport with the patient - Respects the patient’s own views, concerns and perceptions |
| **Preparation** | - Ensures all appropriate pre-procedure checks are performed as per local policies - Ensures that all assisting staff are fully appraised of the current case - Ensures that all medications and accessories likely to be required for this case are available |
| **Equipment Check** | - Ensures the available scope is appropriate for the current patient. - Ensures the endoscope is functioning normally before attempting   insertion checking all channels and connections, light source and angulation locks  are off. |
| **Monitoring** | - Ensures appropriate monitoring of oxygen saturation and vital signs pre- procedure - Ensures appropriate action taken if readings are sub-optimal - Demonstrates awareness of clinical monitoring throughout procedure |
| **Sedation** | - When indicated inserts and secures IV access and uses appropriate topical anaesthesia - Uses sedation and/or analgesic doses in keeping with current guidelines and in the context of the physiology of the patient - Drug doses checked and confirmed with the assisting staff |
| **Insertion and withdrawal** | |
| **Scope handling** | - Exhibits good external control of gastroscope at all times. - Efficient and effective manipulation, using rotation of the head of the scope with the left hand to generate torque and the right hand to insert and withdraw. - Minimizes external looping in shaft of instrument. |
| **Angulation**  **controls** | - Demonstrates ability to use angulation controls appropriately, using the left hand only during the vast majority of the procedure. |
| **Suction/air/lens**  **cleaning** | - Well-judged and timely use of distension, suction and lens clearing. |
| **Tip control** | - Use of torque and angulation wheels independently and in combination, as necessary to elicit excellent controlled tip movement. - Avoids unnecessary mucosal contact, maintaining luminal view when possible. |
| **Intubation and** | - Insertion through the mouth and pharynx under endoscopic vision. |

Formative DOPS_Upper GI (OGD).docx Date Last updated - 01 August 2016

© Royal College of Physicians, London 2016. All rights reserved Page 3 of 5

| **oesophagus** | - Careful and safe intubation of the oesophagus under endoscopic vision. - Passage down the oesophagus under endoscopic vision. |
| --- | --- |
| **Stomach** | - Smooth passage through the stomach and pylorus, maintaining luminal views. - Rapid recognition of all major landmarks. |
| **2^nd^ part of duodenum** | - Insertion into second part of duodenum. - Optimisation of scope position in second part of duodenum. |
| **Pro-active Problem Solving** | - Demonstrates and can articulate a logical approach to resolving technical challenges (bend negotiation, pathology encountered, large hiatus hernia) to ensure complete gastroscopy achieved. - Is able to adapt approach depending on anatomy and technical challenge faced ensuring best option is used. - Early recognition of lack of success of a technique with adaptation or change in strategy to next appropriate potential solution. |
| **Pace and**  **Progress** | - Completes whole procedure in reasonable and appropriate time, without rushing and without unduly prolonging the procedure |
| **Patient comfort** | - Conscious awareness of patient discomfort and potential causes at all times - Applies logical strategy to minimise any potential or induced discomfort, including anticipation of problems and reducing patient anxiety - Appropriate escalation of analgesic use if technical strategies unsuccessful in managing patient discomfort |
| **Visualisation** | |
| **Oesophagus** | - Full and careful visualisation of the whole length of the oesophagus |
| **Gastro- oesophageal junction** | - Correct identification of the both the gastro- oesophageal junction and the squamo-columnar junction. - Full views of gastro-oesophageal junction from both proximally and distally*.* |
| **Fundus** | - Full visualisation of all areas of the gastric fundus with retrograde viewing |
| **Lesser curve** | - Full visualisation of whole length of lesser curve using antegrade and retrograde viewing |
| **Greater curve** | - Full visualisation of whole length of greater curve using antegrade and retrograde viewing |
| **Incisura** | - Full visualisation of proximal and distal margins of the incisura |
| **Antrum and**  **pylorus** | - Full visualisation of the antrum, pylorus and pyloric channel |
| **1^st^ part**  **duodenum** | - Full and careful visualisation of all walls of the 1^st^ part of the duodenum |
| **2^nd^ part**  **duodenum** | - Careful visualisation of distal duodenum |
| **Management of Findings** | |
| **Recognition** | - Rapid, accurate and thorough determination of normal and abnormal findings. - Appropriate use of mucosal enhancement techniques. |
| **Management** | - Takes appropriate specimens as indicated by the pathology and clinical context. - Full and appropriate attempt to visualise important associated lesions. - Performs endoscopic therapy or interventions appropriately for the pathology   and clinical context (includes taking no action) |
| **Complications** | - Ensures the risk of complications is minimised - Rapid recognition of complications both during and after the procedure. - Manages any complications appropriately and safely. |
| **Post procedure** | |
| **Report writing** | - Records a full and accurate description of procedure and findings - Uses appropriate endoscopy scoring systems |
| **Management** | - Records an appropriate management plan (including medication, further |

Formative DOPS_Upper GI (OGD).docx Date Last updated - 01 August 2016

© Royal College of Physicians, London 2016. All rights reserved Page 4 of 5

| **plan** | investigation and responsibility for follow-up). |
| --- | --- |
| **ENTS (endoscopic non-technical skills)** | |
| **Communication and teamwork** | - Maintains clear communication with assisting staff - Gives and receives knowledge and information in a clear and timely fashion - Ensures that both the team and the endoscopist are working together, using the same core information and understand the ‘big picture’ of the case - Ensures that the patient is at the centre of the procedure, emphasising safety and comfort - Clear communication of results and management plan with patient and/or carers |
| **Situation awareness** | - Ensure procedure is carried out with full respect for privacy and dignity - Maintains continuous evaluation of the patient's condition - Ensures lack of distractions and maintains concentration, particularly during difficult situations - Intra-procedural changes to scope set-up monitored and rechecked |
| **Leadership** | - Provides emotional and cognitive support to team members by tailoring leadership and teaching style appropriately - Supports safety and quality by adhering to current protocols and codes of clinical practice - Adopts a calm and controlled demeanour when under pressure, utilising all resources to maintain control of the situation and taking responsibility for patient outcome |
| **Judgement and decision making** | - Considers options and possible courses of action to solve an issue or problem, including assessment of risk and benefit - Communicates decisions and actions to team members prior to implementation - Reviews outcomes of procedure or options for dealing with problems - Reflects on issues and institutes changes to improve practice |

Formative DOPS_Upper GI (OGD).docx Date Last updated - 01 August 2016

© Royal College of Physicians, London 2016. All rights reserved Page 5 of 5
